# Preoperative Inflammatory Markers as a Predictor of Three-Year Overall Survival in Older Cancer Patients Undergoing Oncologic Surgery

**DOI:** 10.3390/cancers13081824

**Published:** 2021-04-11

**Authors:** Baukje Brattinga, Abraham Rutgers, Jacco J. De Haan, Anthony R. Absalom, Hanneke van der Wal-Huisman, Geertruida H. de Bock, Barbara L. van Leeuwen

**Affiliations:** 1 Department of Surgery, University Medical Center Groningen, University of Groningen, 9700 RB Groningen, The Netherlands; b.brattinga@umcg.nl (B.B.); h.van.der.wal-huisman@umcg.nl (H.v.d.W.-H.); 2 Department of Rheumatology and Clinical Immunology, University Medical Center Groningen, University of Groningen, 9700 RB Groningen, The Netherlands; a.rutgers@umcg.nl; 3 Department of Medical Oncology, University Medical Center Groningen, University of Groningen, 9700 RB Groningen, The Netherlands; j.j.de.haan@umcg.nl; 4 Department of Anesthesiology, University Medical Center Groningen, University of Groningen, 9700 RB Groningen, The Netherlands; a.r.absalom@umcg.nl; 5 Department of Epidemiology, University Medical Center Groningen, University of Groningen, 9700 RB Groningen, The Netherlands; g.h.de.bock@umcg.nl

**Keywords:** preoperative inflammatory markers, inflammatory response, survival, older patients, cancer

## Abstract

**Simple Summary:**

Older patients can have an increased expression of inflammatory cytokines due to ageing of the immune system. It is likely that older cancer patients with a low-grade inflammatory state are more at risk for an exaggerated inflammatory response to surgery and for poor outcome after surgery. The aim of this study was to examine whether preoperative inflammatory markers could be a predictor of overall survival in older patients undergoing oncologic surgery. In this prospective cohort study, a plasma level of C-reactive protein (CRP) ≥ 10 mg/L was a predictor of inferior three-year overall survival after oncologic surgery in older cancer patients, and also for the specific group of older patients with a colorectal tumor. Measuring preoperative plasma level of CRP might be useful in risk stratification for poor outcome after surgery in older cancer patients.

**Abstract:**

Oncologic surgery results in substantially higher morbidity and mortality rates in older patients compared to younger patients, yet little is known about the relation between the preoperative inflammatory state and postoperative outcome in the specific group of older cancer patients. The aim of this study was to examine whether preoperative inflammatory markers could be a predictor of overall survival in older patients undergoing elective surgery for a solid malignant tumor. Patients 65 years and older undergoing surgery for a solid malignant tumor were included in a prospective cohort study. Inflammatory markers C-reactive protein (CRP), interleukin-1 beta (IL-1β), IL-6, IL10, IL-12 and tumor necrosis factor-alpha (TNF-α) were measured in plasma samples preoperatively. The main outcome was overall survival three years after surgery. Between 2010 and 2016, 328 patients with a median age of 71.5 years (range 65–89) were included. A significantly higher mortality rate three years after surgery, was found in patients with high preoperative plasma levels of CRP and IL-6 (*p* = 0.013 and *p* = 0.046, respectively). In multivariate analysis, corrected for variables such as age, disease stage, frailty, comorbidities, type of surgery and complications, a preoperative plasma level of CRP ≥ 10 mg/L was an independent prognostic factor for inferior overall survival three years after surgery (multivariate hazard ratio 1.50, 95% confidence interval 1.04–2.16, *p* = 0.031). Also, for the specific group of patients with colorectal cancer, a preoperative plasma level of CRP ≥ 10 mg/L was a prognostic factor for inferior survival three years after surgery (multivariate hazard ratio 2.40, 95% confidence interval 1.20–4.81, *p* = 0.014). Preoperative elevated plasma level of CRP is an independent unfavorable prognostic factor for overall survival three years after oncologic surgery. This gives more insight into the relationship between inflammation and survival in older cancer patients, and might contribute to risk stratification for poor outcome after surgery in older cancer patients.

## 1. Introduction

As the world-wide population is ageing, the incidence of cancer in older patients is increasing [1]. Surgery is the primary treatment for patients with a solid malignant tumor. With the increasing population of older people with cancer, more patients are in need of oncologic surgery. Surgery for a solid malignant tumor results in substantially higher morbidity and mortality rates in older patients compared to younger patients [2]. In risk stratification, decision-making and in the allocation of resources to prevent and manage complications, it would be helpful to have prognostic factors available to assist in determining which older cancer patients are at an increased risk of a poor outcome.

Ageing-related remodeling of the immune system in older patients results in a low-grade inflammatory state with increased expression of cytokines [3]. Increased cytokine levels are linked to the development and clinical progression of cancer and other age-related diseases [4]. Because of the primed immune system, older patients can have an exaggerated inflammatory response to surgery [5]. It is likely that the combination of preoperatively elevated inflammatory markers and an increased inflammatory response to surgery makes older cancer patients more susceptible to poor outcome after surgery. It is already known that preoperatively elevated cytokine levels in older patients lead to a higher incidence of postoperative complications [6]. As complications increase mortality rates [7], it is plausible that the inflammatory state prior to surgery will influence survival after surgery in older cancer patients.

Biomarkers that might be useful as prognostic factors for survival are C-reactive protein (CRP), interleukin-1β (IL-1β), IL-6, IL-10, IL-12 and tumor necrosis factor alpha (TNF-α) [8]. TNF-α is an early mediator in the inflammatory response. IL-β, IL-6, IL-10 and IL-12 are pro- and anti-inflammatory cytokines that can suppress or up-regulate the inflammatory response. CRP is an acute phase protein produced in response to the presence of inflammatory cytokines and is a standard clinical inflammatory marker [8]. Some studies suggest that preoperative plasma levels of some of the inflammatory markers are related to survival [9,10,11]. The Glasgow Prognostic score (GPS) is a validated tool for predicting survival for several types of cancer using a combination of CRP and albumin levels [12]. However, this tool does not use other inflammatory cytokines that might predict survival as well. It would be interesting to know if other inflammatory markers are also related to survival for a better understanding of the mechanism of inflammation and the relation to survival in older patients. In older patients, it is particularly likely that the inflammatory state will influence survival after surgery. The aim of this study was to examine the relationship between preoperative inflammatory markers and overall survival in older patients undergoing elective surgery for a solid malignant tumor.

## 2. Materials and Methods

### 2.1. The PICNIC and PICNIC-B-HAPPY Study

This follow-up study is a sub-study of the prospective observational cohort studies “PICNIC” (Postoperative Cognitive Dysfunction in Elderly Cancer Patients) and “PICNIC-B-HAPPY” (Predicting Postoperative Outcome in Elderly Surgical Cancer Patients: Biomarkers and Handgrip Strength as Predictors of Postoperative Outcome in the Elderly), conducted at the University Medical Center Groningen (UMCG), The Netherlands. The aim of these two cohort studies was to identify factors predicting postoperative physical and cognitive outcome. These studies are registered in the Dutch Clinical Trial Database at www.trialregister.nl: NL4219 (2010-07-22) and NL4441 (2014-06-01). Both cohort studies were approved by the Medical Ethical Committee of the UMCG. Clinical data such as age, gender, disease stage, tumor type, comorbidities according to the Charlson Comorbidity Index (CCI), frailty according to the Groningen Frailty Indicator (GFI) were prospectively collected. Data about the use of medication were collected but not taken into account in analyses in this study. Duration of anesthesia and blood loss were measured perioperatively. Complications following surgery were collected up to 30 days postoperatively, defined according to the Clavien Dindo Classification. Surgery was performed between August 2010 and December 2016. Patients from the PICNIC cohort were earlier described [5,6,13,14], as well as the patients from the PICNIC-B-HAPPY cohort [15,16].

### 2.2. Patients—Inclusion and Exclusion Criteria

In this follow-up study, patients aged 65 years and older undergoing surgery for a solid malignant tumor were included from both cohort studies. Patients were excluded from the subsequent analysis if histological examination of the tumor revealed a benign tumor, or in the case of an incomplete set of inflammatory markers determined preoperatively.

### 2.3. Blood Sampling and Biochemical Analysis

In plasma collected preoperatively, before anesthesia induction, levels of the following inflammatory biomarkers were determined: CRP (Lower limit of detection (LLD): 0.001 μg/mL, Intra-assay coefficient of variation (CV-intra): 5.93%, Inter-assay coefficient of variation (CV-inter): 6.92%), IL-1β (LLD: 1.27 pg/mL, CV-intra: 6.79%, CV-inter: 6.05%), IL-6 (LLD: 0.00 pg/mL, CV-intra: 3.33%, CV-inter: 6.30%), IL-10 (LLD: 3.28 pg/mL, CV-intra: 5.40%, CV-inter: 15.73%), IL-12 (LLD: 5.07 pg/mL, CV-intra: 3.41%, CV-inter: 13.32%), and TNF-α (LLD: 6.49 pg/mL, CV-intra: 5.81%, CV-inter: 6.94%). Analyses of the plasma samples were performed in batches by Haemoscan^®^ (Groningen, The Netherlands) by sandwich ELISA (Enzyme-Linked Immuno Sorbent Assay) for interleukins and TNF-α (BioLegend, San Diego, CA, USA), and high-sensitivity CRP ELISA for CRP (Dakopatts, Glostrup, Denmark). In the analysis of preoperative interleukins and TNF-α, the upper quartile was chosen as a cutoff between patients with high and low plasma levels. This cutoff was chosen based on earlier studies [17,18]. For CRP, a plasma level of 10 mg/L was selected as a cutoff level, as higher levels indicate active inflammation [19,20].

### 2.4. Outcomes

The primary endpoint was overall survival at three years after surgery for older patients with cancer. The secondary outcome was overall survival at three years after surgery for the specific group of older patients with colorectal cancer. Three-year survival was determined for all included patients. Survival data were collected by using electronic health records and missing survival data were collected using the Key Register of Persons.

### 2.5. Data Analysis and Statistics

Normality of continuous data was visually inspected by conducting histograms. Demographic data were described in percentages, medians and ranges. Survival was estimated by Kaplan–Meier and survival curves. Univariate and multivariable Cox regression analyses were performed and hazard ratios (HRs) and the corresponding 95% confidence intervals (95% CIs) were estimated. The log-rank test was used to compare Kaplan–Meier survival curves. *p*-values < 0.05 were considered to be statistically significant. Data analysis was performed using IBM SPSS version 23 (IBM Corporation, Armonk, NY, USA).

## 3. Results

**Figure 1:**
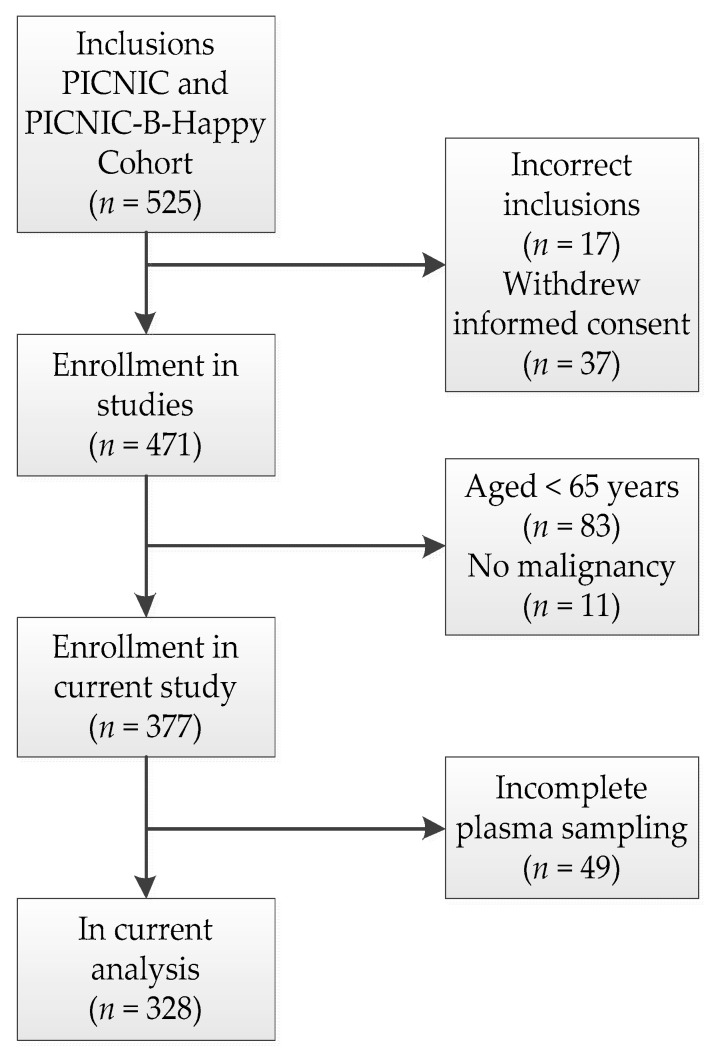
Study flowchart.

A total of 328 patients were included in the current analysis (Figure 1). The median age was 71.5 years (range 65–89) and 178 (54%) patients were male (see Table 1). The most prevalent type of cancer was colorectal cancer (31%), and the majority of all included patients had a carcinoma (71%). A summary of the patient characteristics classified according to whether or not they survived until 3 years after surgery is shown in Table 1. A specification of the different tumor locations is included in the Appendix A (Table A1). Patients who survived more than 3 years after surgery had higher mean plasma levels of the inflammatory cytokines, except for IL-1β and CRP (Table 1). A wider range in plasma levels for patients in the alive group could explain the higher mean plasma levels measured for the patients who survived more than 3 years after surgery.

### 3.1. Survival

**Figure 2:**
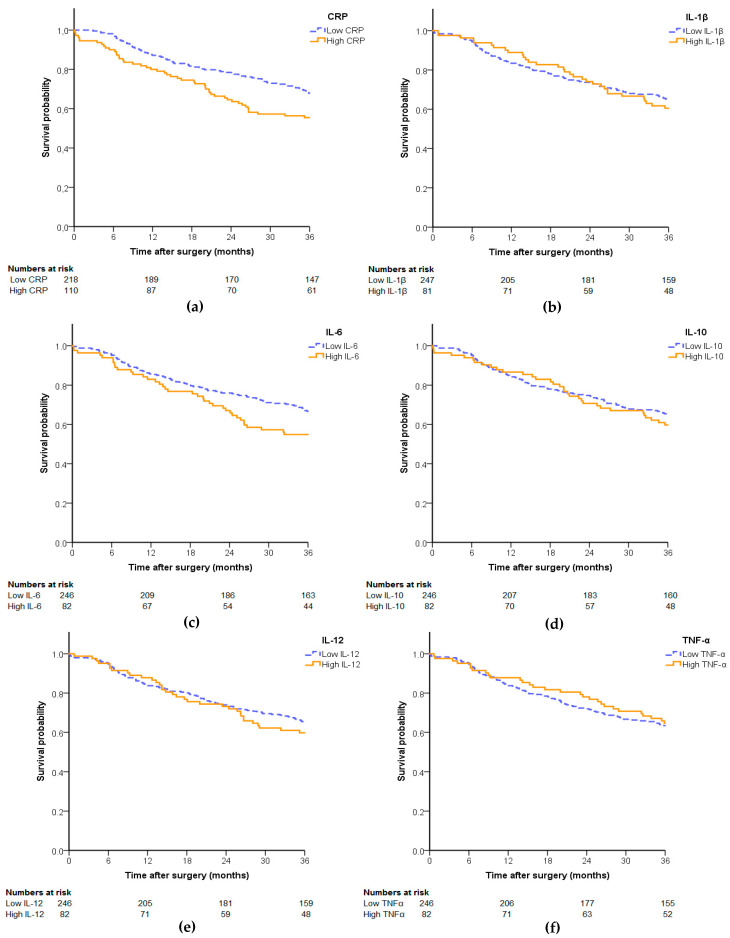
Kaplan–Meier curves displaying survival rates at 3 years after surgery among patients dichotomized as having high ^1^ or low ^2^ preoperative levels of the inflammatory markers: (**a**) CRP, (**b**) IL-1β, (**c**) IL-6, (**d**) IL-10, (**e**) IL-12 and (**f**) TNF-α. ^1^ High is defined as being in the highest 25% of plasma levels for interleukins and TNF-α, and ≥10 mg/L for CRP. ^2^ Low is defined as being in the lowest 75% of plasma levels for interleukins and TNF-α, and <10 mg/L for CRP.

One-year overall survival was 85%, two-year overall survival was 74% and three-year overall survival was 64%. Figure 2 shows the estimated overall survival probability after surgery for all preoperative inflammatory markers. Patients with a higher preoperative plasma level of CRP and IL-6 had a significantly higher mortality rate (*p* = 0.013 and *p* = 0.046, respectively).

### 3.2. Inflammatory Markers—All Included Patients

With Cox regression analyses, the relation between the preoperative inflammatory biomarkers and overall survival was examined. The variables age, gender, comorbidities, frailty, disease stage, tumor type, neo-adjuvant therapy, type of surgery, type and duration of anesthesia and complications were used as possible confounders. Univariate analysis showed that a high level of CRP (≥10 mg/L) was significantly associated with higher risk of mortality (HR 1.58 (CI 1.10–2.28), *p* = 0.014). A high level of IL-6 was also related to an increased risk of mortality (HR 1.48 (CI 1.00–2.18), *p* = 0.048) (Table 2).

A multivariate Cox regression model showed that disease stage (HR 3.79 (CI 2.10–6.85), *p* < 0.001), postoperative complications (HR 1.81 (CI 1.15–2.84), *p* = 0.010) and CRP (HR 1.50 (CI 1.04–2.16), *p* = 0.031) are independent predictors of overall survival three years after surgery (Table 2). Compared with patients with a low CRP (<10 mg/L), patients with a high CRP (≥10 mg/L) had a 50% higher risk of mortality. No significant hazard ratios were observed for the other inflammatory markers. An overview of mean CRP levels for patients within the same disease stage is added in the Appendix A (Table A3).

### 3.3. Inflammatory Markers—Patients with Colorectal Cancer

For the subgroup of patients with colorectal cancer, a Cox regression analysis was made to examine the relation between preoperative inflammatory markers and overall survival for this specific tumor type. The variables age, gender, comorbidities, frailty, disease stage, neo-adjuvant therapy, duration of anesthesia and complications were used as possible confounders. In univariate analysis, high preoperative plasma levels of CRP (HR 2.01 (CI 1.01–3.98), *p* = 0.047), IL-6 (HR 2.05 (CI 1.02–4.13), *p* = 0.044) and IL-12 (HR 2.21 (CI 1.11–4.39), *p* = 0.023) were related to inferior overall survival three years after surgery (Table 3). Multivariate analysis showed that CRP (HR 2.40 (CI 1.20–4.81), *p* = 0.014), disease stage (HR 3.94 (CI 1.89–8.22), *p* < 0.001) and postoperative complications (HR 3.54 (CI 1.64–7.64), *p* = 0.001) are independent significant predictors of overall survival three years after surgery (Table 3).

## 4. Discussion

In this follow-up study, the relationship between preoperative plasma levels of inflammatory markers and survival in older cancer patients was examined. The data show that the preoperative plasma level of CRP was an independent significant prognostic factor for overall survival up to three years after surgery in older patients with cancer. For the specific group of older patients with colorectal cancer, one of the most common cancers diagnosed, the preoperative plasma level of CRP was also an independent prognostic factor for overall survival up to three years after surgery.

Several other studies already identified a preoperatively elevated plasma level of CRP as a prognostic factor for survival after surgery in cancer patients for different types of cancer. In a study with 525 patients with colon cancer with a median follow-up time of 4.5 years, Kersten et al. found that a preoperative CRP of >30 mg/L was associated with inferior disease specific survival (HR 2.1 95% CI (1.39–3.10)). Although the median age in that study was 73, the included patients were not specifically old (range 31–97) [11]. Hrab et al. investigated 89 patients with renal cell carcinoma (mean age 60 years (range 30–81)), and found that elevated preoperative plasma level of CRP was also correlated with impaired overall survival and disease-specific survival within 6 years after surgery. However, multivariate Cox regression analyses were not performed to adjust for possible confounders [21]. Lopez-Pastorini et al. found that in a large study of 1349 patients with lung cancer (mean age <65 years), a preoperative CRP >40 mg/L was an independent significant indicator for inferior survival within 30 days after surgery [22]. Our current study shows that, for the specific group of older cancer patients in need of oncologic surgery, an elevated plasma level of preoperative CRP of only ≥10 mg/L is an unfavorable independent prognostic factor for overall survival after surgery. The plasma level of CRP that is associated with inferior survival in the current study is lower compared to the studies mentioned above. The current study only included older cancer patients, a distinct patient group when it comes to challenges for optimal treatment choice.

The underlying mechanism that explains the link between elevated preoperative plasma level of CRP and impaired overall survival in older patients is still not fully understood. Remodeling of the immune system in older patients is a possible explanation for increased inflammatory markers. The continuous attrition of the immune system through lifelong antigenic stress by antigens, oxidative stress, clinical and subclinical infections leads to chronic activation and remodeling of the immune system. This age-related process of senescence of the immune system is characterized by the production and accumulation of less effective memory and effector T cells. These changes in the immune system cause a decline in reliability and efficiency of the immune response to antigens, leading to chronic increased plasma levels of inflammatory cytokines [23,24]. Chronic inflammatory activity can eventually lead to tissue degeneration because of an increased release of reactive oxygen species (ROS) [25]. In patients with cancer, tumor itself and neo-adjuvant treatment like chemotherapy can also be a source of chronic inflammatory activity. This fact makes it more likely that patients with a severe disease stage have higher inflammatory markers [26]. Chronic inflammation is associated with the development and clinical progression of age-related diseases like cancer, and degenerative processes causing physical and cognitive decline. In this way, chronic inflammation contributes to accelerated ageing, and is associated with high all-cause morbidity and mortality rates [27].

Ageing can have dual effects on the immunological responses of elderly people. Older cancer patients may be less able to respond to new antigens (such as pathogens), but they can also have an exaggerated inflammatory response to surgery-induced injury to their own tissues [5], both contributing to more frequent and severe postoperative complications [2,6]. It seems likely that older cancer patients who are in a chronic inflammatory state, apparent from elevated levels of preoperative inflammatory markers, are at an increased risk for an exaggerated inflammatory response to surgery, postoperative complications, and the associated inferior outcome.

Although preoperative CRP was found to be a predictor for overall survival, no significant relation was found between preoperative levels of the inflammatory cytokines IL-1β, IL-6, IL-10, IL-12 and TNF-α and survival. The cytokines that were examined have different roles in the inflammatory response. IL-1β, IL-6 and TNF-α are pro-inflammatory cytokines that are involved in the acute and chronic inflammatory response. TNF-α regulates the release of pro- and anti-inflammatory cytokines. An imbalance between pro- and anti-inflammatory cytokines can be the driving force of ageing of the immune system [28]. IL-12 has an antitumor and an immune modulatory role and is suppressed by IL-10, an anti-inflammatory cytokine [29]. In the current literature, there is limited evidence for an association between preoperative elevated levels of interleukins and overall survival. One study discovered that high preoperative levels of IL-12 were a prognostic factor for superior overall survival rate in patients with colorectal cancer [30]. Other studies found an association between IL-6 and overall survival in patients with T2 gallbladder cancer and esophageal cancer [10,31]. IL-6 has also been found to be a predictor for the onset of negative health-related events in older nondisabled patients [32]. Because CRP is produced in response to especially IL-6, a relation between increased IL-6 and survival was also expected in the current study. This lacking correlation could be caused by the fact that CRP production in the liver is also stimulated by IL-1 and TNF [19]. CRP has many different functions including promotion of phagocytosis, apoptosis, the production of cytokines and the recruitment of circulating leukocytes to areas of inflammation. Also, smooth muscle cells, macrophages and adipocytes can produce CRP [33]. It is known that IL-6 and CRP have a half-life time of more than 10 h, and TNF-α has a half-life time of 5–10 min. Some of the cytokines are more locally active and are more difficult to measure in plasma samples. These differences in etiology, function and plasma half-life may lead to differences in preoperative plasma levels of CRP and other inflammatory markers [34,35,36].

Overall survival after oncologic surgery is influenced by many patient- and tumor-related factors that are known preoperatively, like age, frailty, comorbidities and disease stage [6,37]. In contrast to the literature, no relation was found between frailty and overall survival, nor between comorbidities and overall survival in older oncologic patients [38]. In this study, only 68 patients were classified as frail based on their GFI score. The lack of association might be because the frailest patients with higher comorbidity levels were more likely to be unfit for surgical treatment or to drop out of the study before surgery [39].

### 4.1. Evaluation of the Study

This is the first study that describes the relationship between plasma levels of different preoperative inflammatory biomarkers and survival for a large group of older cancer patients with different types of cancer. Most of the inflammatory cytokines have not been studied previously in older cancer patients in a preoperative setting. Even in a heterogeneous group of older cancer patients with different types of cancer and different disease stages, CRP is still a significant prognostic factor of overall survival. However, this study also has some limitations. In this study, it was not known if the included patients had an active infection at the time of determining plasma levels. For a better understanding of the inflammatory response, it would be interesting to know if patients with preoperatively elevated plasma levels of CRP had a clinical infection prior to surgery, and if the cause of death is related to inflammatory complications. Further evaluation of the perioperative inflammatory response is necessary for a better understanding of the underlying mechanism. Other inflammatory markers with a pro-inflammatory function like the enzyme cyclooxygenase-2 (COX-2) can also be potential markers in the inflammatory response following surgery. Further research is necessary for a better understanding of the role of other inflammatory markers in relation to survival after surgery. The effect of the presence of comorbidities leading to an overactive immune system, and the use of immune suppressive medication, were not analyzed in this study but might be interesting for further evaluation.

### 4.2. Further Perspectives and Clinical Implementations

CRP is already a frequently used marker in clinical practice and an easy and relatively cheap measurement. This offers possibilities in implementing plasma level of CRP as a preoperative measurement in a risk stratification algorithm. Besides age, frailty and disease stage, a preoperative elevated plasma level of CRP might help in detecting older cancer patients who are at risk for poor outcome after surgery. For implementing preoperative plasma level of CRP in a risk stratification model, further research is necessary to calculate positive and negative predictive values from all possible risk factors for poor survival. Further research of the perioperative inflammatory response is needed to determine if anti-inflammatory drugs could be beneficial in preoperative treatment of older cancer patients in need of oncologic surgery.

## 5. Conclusions

Our data have revealed an association between preoperative plasma level of CRP and overall survival. Preoperative plasma level of CRP could be considered for implementation in a risk stratification model for poor outcome after oncologic surgery. Further research of the inflammatory response perioperatively is necessary to understand the underlying process of inflammation, and also further prospective research for the implementation of preoperative plasma level of CRP in a risk stratification model is needed to validate our findings.

## Figures and Tables

**Table 1:** Patient and tumor characteristics (*n* = 328).

Patient and Surgical Characteristics	Overall (*n* = 328)	Alive >3 Years after Surgery (*n* = 209)	Deceased ≤3 Years after Surgery (*n* = 119)
Age, mean (SEM), y	72.7 (0.3)	72.4 (0.4)	73.3 (0.5)
Gender, No. (%)			
Female	150 (46)	97 (65)	53 (35)
Male	178 (54)	112 (63)	66 (37)
BMI ^a^, No. (%)	-	-	-
<30 kg/m^2^	262 (80)	167 (64)	95 (36)
≥30 kg/m^2^	66 (20)	42 (64)	24 (36)
Charlson comorbidity index (CCI), mean (SEM) *	4 (0.1)	4 (0.1)	4 (0.2)
Groningen Frailty Indicator (GFI), mean (SEM)	2 (0.1)	2 (0.1)	3 (0.2)
Tumor Location, No. (%)			
Colorectal	101 (31)	68 (67)	33 (33)
Gastroesophageal	44 (13)	24 (55)	20 (45)
Gynecological	54 (16)	37 (69)	17 (31)
Skin	42 (13)	28 (67)	14 (33)
Soft tissue	39 (12)	26 (67)	13 (33)
Other	48 (15)	26 (54)	22 (46)
Tumor type, No. (%)			
Carcinoma	233 (71)	144 (62)	89 (38)
Sarcoma	34 (10)	24 (71)	10 (29)
Melanoma	35 (11)	24 (69)	11 (31)
Other malignancy	26 (8)	17 (65)	9 (35)
Disease stage, No. (%)			
I	79 (24)	64 (81)	15 (19)
II	85 (26)	62 (73)	23 (27)
III	89 (27)	49 (55)	40 (45)
IV	75 (23)	34 (45)	41 (55)
Neo-adjuvant treatment, No. (%)			
None	233 (71)	152 (65)	81 (35)
Chemotherapy	30 (9)	16 (53)	14 (47)
Radiation	20 (6)	16 (80)	4 (20)
Combination	45 (14)	25 (56)	20 (44)
Biomarkers T0, mean (SEM)			
CRP ^b^ (mg/L)	12.7 (1.2)	11.4 (1.6)	14.9 (2.0)
IL ^c^-1β (pg/mL)	14.5 (5.4)	14.0 (7.2)	15.4 (7.9)
IL-6 (pg/mL)	27.6 (8.5)	34.7 (13.1)	15.2 (4.4)
IL-10 (pg/ mL)	29.0 (7.1)	32.8 (10.8)	22.2 (5.2)
IL-12 (pg/mL)	13.1 (6.8)	18.5 (10.6)	3.8 (2.3)
TNF ^d^-α (pg/mL)	46.1 (17.3)	54.8 (26.2)	30.8 (11.8)
Type of surgery, No. (%)			
Intracavitary (thorax/abdomen)	229 (70)	136 (59)	93 (41)
Extremities/superficial	99 (30)	73 (74)	26 (26)
Type of anesthesia, No. (%)			
Regional	10 (3)	8 (80)	2 (20)
General	153 (47)	99 (65)	54 (35)
Regional + general	165 (50)	102 (62)	63 (38)
Duration of anesthesia in minutes, mean (SEM)	262 (9)	250 (11)	283 (18)
Postoperative complications, No. (%)			
Clavien Dindo ≥3	45 (14)	21 (47)	24 (53)
Clavien Dindo <3	283 (86)	188 (66)	95 (34)

Variables are denoted as mean and standard error of mean (SEM) or as percentages. ^a^ Body mass index, ^b^ C-reactive protein, ^c^ Interleukin, ^d^ Tumor necrosis factor. * A specification of all the different comorbidities is included in Table A2 in the Appendix A.

**Table 2:** Baseline variables related to overall survival until three years after surgery (univariate and multivariate Cox regression analysis; *n* = 328).

Covariates		Univariate Model HR ^a^ (95% CI) ^b^	Multivariate Model (Overall) HR ^a^ (95% CI) ^b^
Biomarkers ^c^ T0			
CRP	<10 mg/L	Ref.	Ref.
-	≥10 mg/L	1.58 (1.10–2.28) *	1.50 (1.04–2.16) *
IL-1β	<0.9 pg/mL	Ref.	-
-	≥0.9 pg/mL	1.10 (0.73–1.65)	-
IL-6	<4.2 pg/mL	Ref.	-
-	≥4.2 pg/mL	1.48 (1.00–2.18) *	-
IL-10	<19.8 pg/mL	Ref.	-
-	≥19.8 pg/mL	1.16 (0.78–1.73)	-
IL-12	<1.2 pg/mL	Ref.	-
-	≥1.2 pg/mL	1.17 (0.79–1.75)	-
TNF-α	<0.9 pg/mL	Ref.	-
-	≥0.9 pg/mL	0.93 (0.61–1.42)	-
Age (years)		1.02 (0.99–1.05)	-
Gender			
Female		Ref.	-
Male		1.11 (0.77–1.59)	-
CCI			
Low (<3)		Ref.	-
High (≥3)		1.44 (0.99–2.08)	-
GFI			
Low (<4)		Ref.	-
High (≥4)		1.50 (0.99–2.26)	-
Tumor Type			
Carcinoma		1.28 (0.85–1.94)	-
Other malignancy		Ref.	-
Disease Stage			
I		Ref. ***	Ref. ***
II		**1.51 (0.79–2.90)**	**1.50 (0.78–2.88)**
III		**2.77 (1.53–5.02)**	**2.63 (1.45–4.77)**
IV		**3.77 (2.08–6.81)**	**3.79 (2.10–6.85)**
Neo-adjuvant Treatment			
None		Ref.	-
Radiation		1.44 (0.82–2.53)	-
Chemotherapy		0.54 (0.20–1.46)	-
Combination		1.42 (0.87–2.32)	-
Type of surgery			
Intra-abdominal/thoracic		1.69 (1.10–2.61) **	-
Extremities/superficial		Ref.	-
Type of anesthesia			
Regional		Ref.	-
General		1.99 (0.48–8.15)	-
Regional + general		2.30 (0.56–9.40)	-
Duration anesthesia (min)			
≤180 min		Ref.	-
>180 min		1.04 (0.72–1.50)	-
Postoperative complications			
Clavien Dindo < 3		Ref.	Ref.
Clavien Dindo ≥ 3		1.96 (1.25–3.06) **	1.81 (1.15–2.84) *

* *p* < 0.05, ** *p* < 0.01, *** *p* < 0.001 (bold values are considered significant). ^a^ Hazard Ratio, ^b^ 95% confidence interval, ^c^ the groups with higher plasma levels contain the highest 25% of plasma levels for interleukins and TNF-α, and ≥10 mg/L for CRP. The groups with lower plasma levels contain the lowest 75% of plasma levels for interleukins and TNF-α and <10 mg/L for CRP.

**Table 3:** Baseline variables related to overall survival until three years after surgery for patients with a colorectal tumor (univariate and multivariate Cox regression analysis; *n* = 101).

Covariates		Univariate Model HR ^a^ (95% CI) ^b^	Multivariate Model (Overall) HR ^a^ (95% CI) ^b^
Biomarkers ^c^ T0		-	-
CRP	<10 mg/L	Ref.	Ref.
-	≥10 mg/L	2.01 (1.01–3.98) *	2.40 (1.20–4.81) *
IL-1β	<0.9 pg/mL	Ref.	-
-	≥0.9 pg/mL	1.68 (0.81–3.46)	-
IL-6	<4.2 pg/mL	Ref.	-
-	≥4.2 pg/mL	2.05 (1.02–4.13) *	-
IL-10	<19.8 pg/mL	Ref.	-
-	≥19.8 pg/mL	0.90 (0.42–1.94)	-
IL-12	<1.2 pg/mL	Ref.	-
-	≥1.2 pg/mL	2.21 (1.11–4.39) *	-
TNF-α	<0.9 pg/mL	Ref.	-
-	≥0.9 pg/mL	1.54 (0.76–3.14)	-
Age (years)		1.01 (0.95–1.08)	-
Gender			
Female		Ref.	-
Male		1.12 (0.54–2.36)	-
CCI			
Low (<3)		Ref.	-
High (≥3)		1.60 (0.74–3.45)	-
GFI			
Low (<4)		Ref.	-
High (≥4)		0.37 (0.09–1.53)	-
Disease Stage			
I + II		Ref.	Ref.
III + IV		3.05 (1.50–6.21) **	3.94 (1.89–8.22) ***
Neo-adjuvant Treatment			
None		Ref.	-
Chemotherapy or radiation		0.95 (0.36–2.53)	-
Combination		1.18 (0.52–2.67)	-
Duration anesthesia (min)			
≤180 min		Ref.	-
>180 min		0.54 (0.27–1.10)	-
Postoperative complications			
Clavien Dindo <3		Ref.	Ref.
Clavien Dindo ≥3		2.83 (1.34–5.97) **	3.54 (1.64–7.64) **

* *p* < 0.05, ** *p* < 0.01, *** *p* < 0.001 (bold values are considered significant). ^a^ Hazard Ratio, ^b^ 95% confidence interval, ^c^ The groups with higher plasma levels contain the highest 25% of plasma levels for interleukins and TNF-α, and ≥10 mg/L for CRP. The groups with lower plasma levels contain the lowest 75% of plasma levels for interleukins and TNF-α, and <10 mg/L for CRP.

## Data Availability

The data presented in this study are available on request from the corresponding author. The data are not publicly available due to privacy issues.

## Appendix

**Table A1:** Specification of the different tumor locations of the included patients (*n* = 328).

Tumor Localization	*n* (%)
Colorectal tumor	101 (30.8)
Gastric tumor	20 (6.1)
Esophageal tumor	24 (7.3)
Small bowel tumor	9 (2.7)
Bile duct or gallbladder tumor	8 (2.4)
Pancreatic tumor	14 (4.3)
Breast tumor	2 (0.6)
Thyroid tumor	15 (4.6)
Vulvar tumor	19 (5.8)
Womb tumor	15 (4.6)
Ovarian tumor	20 (6.1)
Skin tumor	42 (12.8)
Soft tissue	39 (11.9)

**Table A2:** Specification of the different comorbidities of the included patients (*n* = 328).

Comorbidities	*n* (%)
Myocardial infarction	16 (4.9%)
Congestive heart failure	33 (10.1%)
Peripheral vascular disease	76 (23.2%)
CVA or TIA	19 (5.8%)
Dementia	1 (0.3%)
COPD	23 (7.0%)
Connective tissue disease	5 (1.5%)
Peptic ulcer disease	3 (0.9%)
Mild liver disease	1 (0.3%)
Moderate/severe liver disease	1 (0.3%)
Diabetes mellitus	45 (13.7%)
Hemiplegia	1 (0.3%)
Chronic kidney disease	6 (1.8%)
AIDS	1 (0.3%)

**Table A3:** CRP levels in patients within the same disease stage (*n* = 328).

Disease Stage, (*n*)	Mean CRP Level in mg/L (SE)
Stage I, (79)	8.9 (1.6)
Stage II, (85)	13.3 (2.7)
Stage III, (89)	14.2 (2.8)
Stage IV, (75)	14.0 (2.3)

## References

[1] Ewertz M., Christensen K., Engholm G., Kejs A.M.T., Lund L., Matzen L.E., Pfeiffer P., Storm H.H., Herrstedt J., on behalf of the Academy of Geriatric Cancer Research (AgeCare) (2016). Trends in cancer in the elderly population in Denmark, 1980–2012. Acta Oncol..

[2] Huisman M., Audisio R., Ugolini G., Montroni I., Vigano A., Spiliotis J., Stabilini C., Carino N.D.L., Farinella E., Stanojevic G. (2015). Screening for predictors of adverse outcome in onco-geriatric surgical patients: A multicenter prospective cohort study. Eur. J. Surg. Oncol. (EJSO).

[3] Vasto S., Candore G., Balistreri C.R., Caruso M., Colonna-Romano G., Grimaldi M.P., Listi F., Nuzzo D., Lio D., Caruso C. (2007). Inflammatory networks in ageing, age-related diseases and longevity. Mech. Ageing Dev..

[4] Davalos A.R., Coppe J.-P., Campisi J., Desprez P.-Y. (2010). Senescent cells as a source of inflammatory factors for tumor progression. Cancer Metastasis Rev..

[5] Plas M., De Haan J.J., Van Der Wal-Huisman H., Rutgers A., Absalom A.R., De Bock G.H., Van Leeuwen B.L. (2019). The systemic impact of a surgical procedure in older oncological patients. Eur. J. Surg. Oncol. (EJSO).

[6] Plas M., Rutgers A., Van Der Wal-Huisman H., De Haan J.J., Absalom A.R., De Bock G.H., Van Leeuwen B.L. (2020). The association between the inflammatory response to surgery and postoperative complications in older patients with cancer; a prospective prognostic factor study. J. Geriatr. Oncol..

[7] Kubota T., Hiki N., Sano T., Nomura S., Nunobe S., Kumagai K., Aikou S., Watanabe R., Kosuga T., Yamaguchi T. (2013). Prognostic Significance of Complications after Curative Surgery for Gastric Cancer. Ann. Surg. Oncol..

[8] Lin E., Calvano S.E., Lowry S.F. (2000). Inflammatory cytokines and cell response in surgery. Surgery.

[9] Szaflarska A., Szczepanik A., Siedlar M., Czupryna A., Sierzega M., Popiela T., Zembala M. (2009). Preoperative plasma level of IL-10 but not of proinflammatory cytokines is an independent prognostic factor in patients with gastric cancer. Anticancer. Res..

[10] Wang J., Liu J., Chang Q., Yang B., Li S., Gu C. (2018). The association between preoperative serum interleukin-6 levels and postoperative prognosis in patients with T2 gallbladder cancer. J. Surg. Oncol..

[11] Kersten C., Louhimo J., Ålgars A., Lahdesmaki A., Cvancerova M., Stenstedt K., Haglund C., Gunnarsson U. (2013). Increased C-reactive protein implies a poorer stage-specific prognosis in colon cancer. Acta Oncol..

[12] McMillan D.C. (2013). The systemic inflammation-based Glasgow Prognostic Score: A decade of experience in patients with cancer. Cancer Treat. Rev..

[13] Plas M., Rotteveel E., Izaks G., Spikman J., van der Wal-Huisman H., van Etten B., Absalom A., Mourits M., de Bock G., van Leeuwen B. (2017). Cognitive decline after major oncological surgery in the elderly. Eur. J. Cancer.

[14] Weerink L.B.M., Van Leeuwen B.L., Msc S.A.M.G., Absalom A.R., Huisman M.G., Msc H.V.D.W.-H., Izaks G.J., De Bock G.H. (2018). Vitamin Status and the Development of Postoperative Cognitive Decline in Elderly Surgical Oncologic Patients. Ann. Surg. Oncol..

[15] Du Msc J., Plas M., Absalom A.R., Leeuwen B.L., De Bock G.H. (2020). The association of preoperative anxiety and depression with neurocognitive disorder following oncological surgery. J. Surg. Oncol..

[16] Bras L., Driessen D.A.J.J., De Vries J., Festen S., Van Der Laan B.F.A.M., Van Leeuwen B.L., De Bock G.H., Halmos G.B. (2019). Patients with head and neck cancer: Are they frailer than patients with other solid malignancies?. Eur. J. Cancer Care.

[17] Amdur R.L., Feldman H.I., Gupta J., Yang W., Kanetsky P., Shlipak M., Rahman M., Lash J.P., Townsend R.R., Ojo A. (2016). Inflammation and Progression of CKD: The CRIC Study. Clin. J. Am. Soc. Nephrol..

[18] Panichi V., Maggiore U., Taccola D., Migliori M., Rizza G.M., Consani C., Bertini A., Sposini S., Perez-Garcia R., Rindi P. (2004). Interleukin-6 is a stronger predictor of total and cardiovascular mortality than C-reactive protein in haemodialysis patients. Nephrol. Dial. Transplant..

[19] Clyne B., Olshaker J.S. (1999). The C-reactive protein. J. Emerg. Med..

[20] Morley J.J., Kushner I. (1982). SERUM C-REACTIVE PROTEIN LEVELS IN DISEASE. Ann. N. Y. Acad. Sci..

[21] Hrab M., Olek-Hrab K., Antczak A., Kwias Z., Milecki T. (2013). Interleukin-6 (IL-6) and C-reactive protein (CRP) concentration prior to total nephrectomy are prognostic factors in localized renal cell carcinoma (RCC). Rep. Pr. Oncol. Radiother..

[22] Lopez-Pastorini A., Riedel R., Koryllos A., Beckers F., Ludwig C., Stoelben E. (2017). The impact of preoperative elevated serum C-reactive protein on postoperative morbidity and mortality after anatomic resection for lung cancer. Lung Cancer.

[23] Ventura M.T., Casciaro M., Gangemi S., Buquicchio R. (2017). Immunosenescence in aging: Between immune cells depletion and cytokines up-regulation. Clin. Mol. Allergy.

[24] De Martinis M., Franceschi C., Monti D., Ginaldi L. (2005). Inflamm-ageing and lifelong antigenic load as major determinants of ageing rate and longevity. FEBS Lett..

[25] Suzuki K. (2019). Chronic Inflammation as an Immunological Abnormality and Effectiveness of Exercise. Biomolecules.

[26] Raposo T., Beirão B., Pang L., Queiroga F., Argyle D. (2015). Inflammation and cancer: Till death tears them apart. Vet. J..

[27] De Martinis M., Franceschi C., Monti D., Ginaldi L. (2006). Inflammation markers predicting frailty and mortality in the elderly. Exp. Mol. Pathol..

[28] Franceschi C., Capri M., Monti D., Giunta S., Olivieri F., Sevini F., Panourgia M.P., Invidia L., Celani L., Scurti M. (2007). Inflammaging and anti-inflammaging: A systemic perspective on aging and longevity emerged from studies in humans. Mech. Ageing Dev..

[29] Minciullo P.L., Catalano A., Mandraffino G., Casciaro M., Crucitti A., Maltese G., Morabito N., Lasco A., Gangemi S., Basile G. (2016). Inflammaging and Anti-Inflammaging: The Role of Cytokines in Extreme Longevity. Arch. Immunol. Ther. Exp..

[30] Stanilov N., Miteva L., Jovchev J., Cirovski G., Stanilova S. (2014). The prognostic value of preoperative serum levels of IL-12p40 and IL-23 for survival of patients with colorectal cancer. APMIS.

[31] Maeda Y., Takeuchi H., Matsuda S., Okamura A., Fukuda K., Miyasho T., Nakamura R., Suda K., Wada N., Kawakubo H. (2019). Clinical significance of preoperative serum concentrations of interleukin-6 as a prognostic marker in patients with esophageal cancer. Esophagus.

[32] Cesari M., Kritchevsky S.B., Nicklas B., Kanaya A.M., Patrignani P., Tacconelli S., Tranah G.J., Tognoni G., Harris T.B., Incalzi R.A. (2012). Oxidative Damage, Platelet Activation, and Inflammation to Predict Mobility Disability and Mortality in Older Persons: Results From the Health Aging and Body Composition Study. J. Gerontol. Ser. A Boil. Sci. Med. Sci..

[33] Sproston N.R., Ashworth J.J. (2018). Role of C-Reactive Protein at Sites of Inflammation and Infection. Front. Immunol..

[34] Takata S., Wada H., Tamura M., Koide T., Higaki M., Mikura S.-I., Yasutake T., Hirao S., Nakamura M., Honda K. (2011). Kinetics of c-reactive protein (CRP) and serum amyloid A protein (SAA) in patients with community-acquired pneumonia (CAP), as presented with biologic half-life times. Biomarkers.

[35] Kuribayashi T. (2018). Elimination half-lives of interleukin-6 and cytokine-induced neutrophil chemoattractant-1 synthesized in response to inflammatory stimulation in rats. Lab. Anim. Res..

[36] Flick D.A., E Gifford G., Glfford G.E. (1986). Pharmacokinetics of Murine Tumor Necrosis Factor. Immunopharmacol. Immunotoxicol..

[37] Fagard K., Leonard S., Deschodt M., Devriendt E., Wolthuis A., Prenen H., Flamaing J., Milisen K., Wildiers H., Kenis C. (2016). The impact of frailty on postoperative outcomes in individuals aged 65 and over undergoing elective surgery for colorectal cancer: A systematic review. J. Geriatr. Oncol..

[38] Bouillon K., Kivimaki M., Hamer M., Sabia S., I Fransson E., Singh-Manoux A., Gale C.R., Batty G.D. (2013). Measures of frailty in population-based studies: An overview. BMC Geriatr..

[39] Mody L., Miller D.K., McGloin J.M., Freeman M., Marcantonio E.R., Magaziner J., Studenski S. (2008). Recruitment and Retention of Older Adults in Aging Research. J. Am. Geriatr. Soc..

